# Research and Remedies

**Published:** 1916-03-25

**Authors:** 


### RESEARCH AND REMEDIES.

### Galyl.

This is an arsenical compound of French origin and described as possessing all the therapeutic properties attributed to salvarsan. A solution of it in sodium carbonate is readily obtained, and the drug is issued in a sealed flask, together with the quantity of the alkali necessary for its solution. Hence it is readily prepared for administration, the volume produced is small, and it is readily introduced into a vein direct from the syringe. Care, however, must be taken to see that the needle is actually in the vein, for galyl introduced into the subcutaneous tissues is, like salvarsan, an irritant, and may produce sloughing. Captain Arthur Foerster has published a series of cases of syphilis with very pronounced lesions and treated by galyl, and he expresses himself as well pleased with the results.—*Lancet.*

### Posterior Pituitary Therapeutics (Pituitrin).

Some very confident and comprehensive claims are made for the virtues of this substance. It is said to raise blood pressure in asthenic conditions and to lower it in hypersthenic states; to act as a diuretic but to control polyuria; to stimulate growth in youth and to delay the decay of old age; to arrest menorrhagia and to cure amenorrhœa; to promote uterine contraction in labour and to subdue after-pains. The suggestion is made that the secretion of the pituitary body acts on all the other endocrinous glands, making these active or quiescent according to the requirements of the organism, these requirements being communicated to the pituitary body through the agency of the nervous system. In ancient times the pituitary body was regarded as the seat of the soul, and modern doctrine would appear to be reverting to this creed. After fourteen years' experience and the treatment of more than a thousand cases Dr. R. Alexander Bute concludes that pituitary substance is the "greatest addition to modern materia medica." He enumerates a long list of diseases in which he has found it useful. They range from acromegaly to tonsillitis, and from intermittent claudication to senile pruritus. Quoting some individual experiences, we note Addison's disease with sphygmometer record raised and granular kidney with a fall of pressure. Much more startling is the statement that in cardiac valvular disease the murmurs "in some cases" cease to be detectable. A case of ulcerative endocarditis following otitis media was saved, and two cases of apoplexy with "death-like" pallor recovered. For exophthalmic goitre the pituitary substance is recommended, together with quinine hydrobromate and the local use of biniodide of mercury. Chronic gout in a man of eighty years has been kept at bay, and in cases of optic neuritis "pronounced hopeless by the ophthalmologist" the results have been amazing. These statements obviously require confirmation by other observers, and it would be well that the pituitary extract were made the subject of exact and careful observation.—*Louisville Journal of Medicine and Surgery.*

### Painless Childbirth.

Dr. J. W. Ballantyne, after an elaborate review of various articles dealing with this subject, concludes that it is not clear that anything has been accomplished to warrant the extravagant statements which have been so freely made in the medical and lay Press. Indirectly the discussion may have done good in drawing attention to the amount of pain suffered in the first stage of labour, and to the need for taking steps to lessen this. Some extension of relief in this direction may be possible, but it is not yet demonstrated that the combination of scopolamine and morphine successfully avoids the dangers of these two drugs when given separately. It is satisfactory to have the verdict of so competent an authority, for highly picturesque statements have been made on the subject in the lay Press both here and in the United States. A book dealing with this subject and written by a lay woman was reviewed in The Hospital on May 8, 1915.—*Edinburgh Medical Journal.*

